# Chimney Detection Based on Faster R-CNN and Spatial Analysis Methods in High Resolution Remote Sensing Images

**DOI:** 10.3390/s20164353

**Published:** 2020-08-05

**Authors:** Chunming Han, Guangfu Li, Yixing Ding, Fuli Yan, Linyan Bai

**Affiliations:** 1 Key Laboratory of Digital Earth Science, Aerospace Information Research Institute, Chinese Academy of Sciences, Beijing 100094, China; hancm@radi.ac.cn (C.H.); ligf@radi.ac.cn (G.L.); yanfl@radi.ac.cn (F.Y.); baily@radi.ac.cn (L.B.); 2 School of Electronic Electrical and Communication Engineering, University of Chinese Academy of Sciences, Beijing 100049, China

**Keywords:** target detection, high resolution remote sensing image, chimney, faster R-CNN, spatial analysis

## Abstract

Spatially location and working status of pollution sources are very important pieces of information for environment protection. Waste gas produced by fossil fuel consumption in the industry is mainly discharged to the atmosphere through a chimney. Therefore, detecting the distribution of chimneys and their working status is of great significance to urban environment monitoring and environmental governance. In this paper, we use an open access dataset BUAA-FFPP60 and the faster regions with convolutional neural network (Faster R-CNN) algorithm to train the preliminarily detection model. Then, the trained model is used to detect the chimneys in three high-resolution remote sensing images of Google Maps, which is located in Tangshan city. The results show that a large number of false positive targets are detected. For working chimney detection, the recall rate is 77.27%, but the precision is only 40.47%. Therefore, two spatial analysis methods, the digital terrain model (DTM) filtering, and main direction test are introduced to remove the false chimneys. The DTM is generated by ZiYuan-3 satellite images and then registered to the high-resolution image. We set an elevation threshold to filter the false positive targets. After DTM filtering, we use principle component analysis (PCA) to calculate the main direction of each target image slice, and then use the main direction to remove false positive targets further. The results show that by using the combination of DTM filtering and main direction test, more than 95% false chimneys can be removed and, therefore, the detection precision is significantly increased.

## 1. Introduction

In recent decades, rapid economic development has led to a significant increase in energy consumption. In China’s primary energy share in 2019, the proportion of fossil energy consumption was still more than 85%, according to the BP Statistical Review of World Energy. The burning of fossil fuels will release a large amount of pollutants into the atmosphere, which will cause serious environmental problems and endanger the health of nearby residents. Among different pollutant discharge sources, the industry discharge contributes the most. The waste gas produced by fossil fuel consumption in industry is mainly discharged to the atmosphere through the chimney. Therefore, the distribution of working chimneys serve as a very important indicator of local air pollution situation. Detecting the number of chimneys and their working status is of great significance to urban environment monitoring and environmental governance.

Target detection on high-resolution remote sensing image provides an efficient and accurate way to detect the position and status of the chimney. There are two types of target detection algorithms: traditional algorithms and algorithms based on deep learning. The traditional algorithms, such as the Local Binary Pattern (LBP) [1] algorithm, scale-invariant feature transform (SIFT) [2] algorithm, and the Support Vector Machine (SVM) [3] algorithm, do not perform well in accuracy and robustness when used for dealing with complex recognition problems [4]. To increase the detection accuracy, a deep learning algorithm, convolutional neural network (CNN) [5], has been proposed to imitate the human brain neuron connection and transfer message mechanism. This kind of deep learning algorithm can be divided into two categories, the one-step algorithm and the two-step algorithm. The one-step algorithm, such as Single Shot MultiBox Detector (SSD) [6], and You Only Look Once (YOLO) [7], has less accuracy as well as lower computational cost. The two-step algorithm, such as region-based convolutional neural networks (R-CNNs) [8], Fast R-CNN [9], and Faster R-CNN [10], is characterized by its high accuracy and high time cost.

At present, deep learning has been successfully applied in remote sensing images in aircraft detection [11,12,13], ship detection [14,15,16], oil tank [17,18,19] detection with good performance. Several experiments on chimney detection have also been reported. Yao et al. [20] used the Faster R-CNN to detect the chimney and condensing tower. Zhang et al. [21] established the BUAA-FFPP60 dataset, which can be used not only to detect the targets, but also to confirm their working status. Comparison among different deep learning algorithms [6,10,22,23,24,25,26,27] is also made based on performance indicators, such as accuracy, model memory size, and running time, and results show that no single algorithm performs well in all aspects. Deng et al. [28] increased the number and scale of feature pyramids, based on the original Feature Pyramid Network (FPN), to improve the detection accuracy.

In practical application, the image always contains various artificial targets. Some targets are very close to the chimney in textures and geometric features, such as roads, building edges, and oil tanks. The Faster R-CNN for chimney detection in the aforementioned references is based on specific datasets that only contain manually selected chimneys. When the Faster R-CNN is used in a large-scale scene, there will be a large number of chimney-like targets that are misclassified into chimneys, leading to a significant decrease in precision. In order to improve the precision, we use two spatial analysis methods. The digital terrain model (DTM) is first introduced. DTM reflects the height fluctuation of ground objects. The chimney is a vertical object and appears elongated in the image. Therefore, where there is a chimney, the DTM will change dramatically. It can be used as a condition to determine whether there is a chimney by detecting the severity of the changes. In addition, in a high-resolution image, the field of view is relatively small, so the changes in observing angle in one image is small. Consequently, the chimneys in one image show the same pointing direction. In this paper, we call this direction the main direction of this image. Therefore, the detected objects that are not in accordance with main direction can be considered as false detections.

In this paper, we use BUAA-FFPP60 dataset [21] and Faster R-CNN algorithm to train the preliminarily detection model. Then, two spatial analysis methods, the DTM filtering and main direction test, are introduced to remove the false chimneys. The detailed description of the method is in Section 2, and the result discussion in Section 3. The results show that the elevation filtering and main direction test are both very effective in reducing false detection rate. Furthermore, the combination of these two methods show extremely good performance in increasing detecting precision.

## 2. Methodology

**Figure 1:**
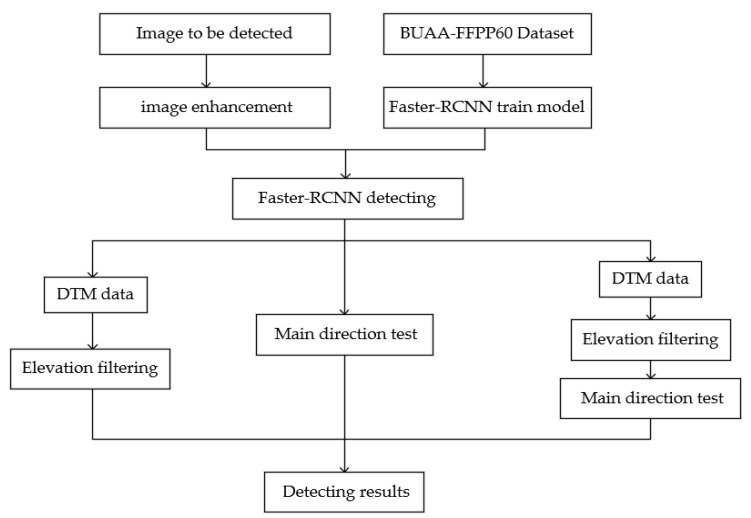
Process diagram.

The method proposed in this paper consists of three parts: (1) the preliminary detection on enhanced images by Faster R-CNN, (2) the elevation filtering using local DTM, (3) the main direction test. The overall process diagram is given in Figure 1. Considering that the condensing tower is detected in former studies, its experimental results are preserved as comparative references. Furthermore, although the thermal infrared data are helpful for detecting the working chimneys, the resolutions of commonly accessible data are too low. Therefore, they are not used in this paper.

### 2.1. Faster R-CNN for Target Detection

**Figure 2:**
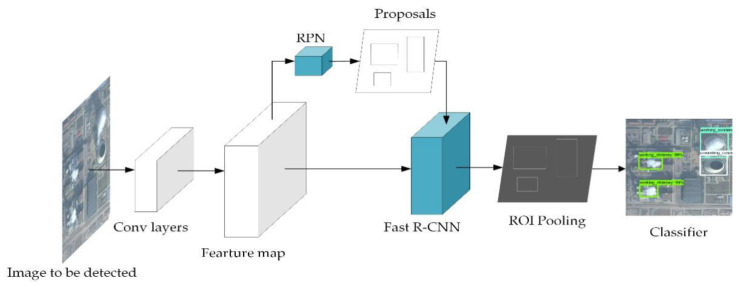
Faster region-based convolutional neural networks (R-CNN) structure diagram.

The Faster R-CNN is chosen for preliminary detection for its high accuracy in chimney detection compared with other methods [21]. As mentioned before, the Faster R-CNN contains two steps [10]. The first step is Region Proposal Network (RPN). RPN takes an image as input and outputs a set of rectangular object proposal regions, each with an objectness score. The second step is Fast R-CNN detection in the proposed regions. Both RPN and Fast R-CNN share the same convolutional layers, rather than learning two separate networks. Figure 2 shows the process structure of Faster R-CNN. It first performs the deep fully convolution on the input image to obtain feature maps. Then, the feature maps are used by RPN to generate proposal regions. Fast R-CNN uses feature map and proposal regions to generate region of interest (ROI) pooling. After that, the fully connected layer is used for classification and regression operations.

Different types of targets correspond to different anchors, which are a serious reference boxes in each sliding-window when region proposals are generated. Anchor size can be obtained from previous experience. In order to fit chimney and condensing tower detection, we set four types anchors of scales: 32^2^, 64^2^, 128^2^, and 256^2^, and five aspect types of ratios: 1:1, 1:2, 1:3, 2:1, and 3:1. The resnet101 [29] trained on coco [30] is selected as the pre-training model. This model is one of widely used model in the field of target detection because of high accuracy and speed.

### 2.2. The Elevation Filtering Using Local DTM

DTM is a digital description of the shape, size, and elevation of terrain. The chimney and condensing tower are usually higher than the surrounding features. In the place where there is a chimney or a condensing tower, the value of DTM shows obvious fluctuations, and the height difference can achieve as large as 20 m. In place where false detection appears, the value of DTM changes more gradually.

To get the DTM slice images, which are pieces of DTM image cut from whole DTM image correspondent to the target bonding box, the detection results of Faster R-CNN are registered to DTM first. Then, the bounding boxes are used to cut several slices from the DTM. Then statistical operations are performed in slices. The max and mean height of each DTM slice are calculated as follows:

(1) $$V_{mean}=\frac{1}{m\times n}\overset{m}{\underset{i=1}{\sum }}\overset{n}{\underset{j=1}{\sum }}f\left(x_{i},y_{j}\right)$$

(2) $$V_{max}=Max\left(f\left(x_{1},y_{1}\right),f\left(x_{2},y_{2}\right)\ldots \;\ldots f\left(x_{m},y_{n}\right)\right)$$

Where *V_mean_* is average value of slice, *V_max_* is the maximum value of slice, *f*(*x_i_, y_j_*) is the pixel value of the slice, m and n are the number of rows and columns of the slice, respectively. The filter condition is given by:

(3) $$\{\begin{array}{c} V_{max}-V_{mean}>T\\ V_{max}-V_{mean}<T \end{array}$$

*T* is threshold value. The difference between the max height and mean height in the slice should be larger than the threshold, or else the detected object will be considered as false positive and removed from the set of detected chimneys. The value of threshold is set to be 20 m according to the National Standard of China, the Emission Standard of air pollutants for boiler [31], in which states that the coal combustion chimney should not be less than 20 m. Moreover, we also experimentally test 5 threshold values. The experiment results are shown in Table 1. When the threshold is 16 m or 18 m, the number of false positive targets is still too large. When the threshold increases to 20 m, although 3 chimneys are mis-removed, the number of false positive targets is greatly reduced. When the threshold is 22 m or 24 m, there will be too many mis-removed chimneys. Thus, a 20 m-threshold seems reach a good compromise between low mis-removal and effective deletion of false positive targets.

### 2.3. Main Direction Test

The chimney is a long and vertical object. In the bounding box, the image slice, which contains a chimney, will show obvious directional texture features. Moreover, the chimney and the condensing tower in one high-resolution remote sensing image are all approximately pointed to the same direction, which is called main direction in this paper. We found that a lot of the mis-detected targets do not have the same feature. Therefore, the false chimneys can be further removed by testing its consistency with the main direction. The principle component analysis (PCA) is used to calculate the main direction of each image slice. The processing flow is:

(1) Gaussian filtering the image slice to remove noise interference;
(2) converting the image slice into a grayscale image;
(3) binarizing and extracting the position coordinates of non-zero pixels to construct a position matrix, and then calculating its covariance matrix;
(4) calculating the eigenvector corresponding to the max eigenvalue of covariance matrix;
(5) calculating the main direction angle of each slice according to the eigenvector.

**Figure 3:**
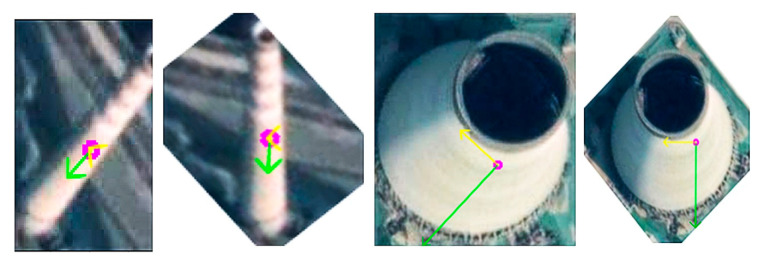
Main direction rotation image. The green arrow represents the main direction, while the yellow arrow represents the direction perpendicular to the main direction.

Figure 3 shows two examples of using this method to find the main direction of each detected target. After calculating the main directions of all slices, the distribution histogram will be mapped at intervals of 5 degrees. The maximum value in the histogram is considered as the main direction *d* of the entire image. Then, the detected target whose main direction is close to the main direction of the image will be considered as true detection. The decision criteria is set to be *d* ± 5° for chimney, and *d* ± 8° for condensing tower since the condensing tower is much wider than the chimney in the image.

## 3. Results

### 3.1. Dataset, Experimental Area, and Data

**Figure 4:**
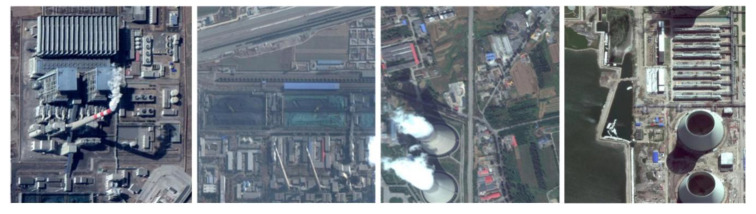
BUAA-FFPP60 dataset samples. Four subfigures indicate the working chimneys, non-working chimneys, working condensing towers, non-working condensing towers, respectively.

The dataset used in this experiment is BUAA-FFPP60, which is collected and produced by Beihang University. The dataset is composed of chimneys and condensation towers distributed in the 123-km^2^ power plant in the Beijing–Tianjin–Hebei area. There are 318 original pictures, of which 31 are test pictures. The remaining 287 pictures are mirrored or rotated by 90° to generate 861 training pictures. The pictures come from Google map with a resolution of 1 m, ranging in size from 500 × 500 to 700 × 1250 pixels. The working state of the chimney and condensation tower is determined by whether there is smoke. The four labels in the dataset are working chimney, non-working chimney, working condensation tower, and non-working condensation tower. Figure 4 shows some examples of dataset.

The area selected for this experiment is Tangshan City, Hebei Province, located 180 km southeast of Beijing. It is a regional core city of Beijing-Tianjin-Tangshan city group, and burdens the task of releasing the industrial pressure of Beijing, the capital of China. Tangshan City is a typical industrial city in North China, and the total crude steel production in 2018 is 133 million tons, about 7.35% of world’s total production. Meanwhile, it is also one of the cities with the worst air quality in the country. According to the “Tangshan City Environmental Status Report”, in 2011, the emissions of sulfur dioxide and nitrogen oxides in Tangshan City were 336.54 thousand tons and 40.59 thousand tons, respectively [32]. Numerous steel factories and power plants with a large number of chimneys and condensation towers in Tangshan have contributed the most to the hazardous air pollutants. Therefore, investigating the position and working status of chimneys and condensation towers is very important to region environmental governance.

Three Google Maps images with 1-m resolution covering about 600 km^2^ are used for final detection. Sizes of images are 16,000 × 25,000 pixels, 10,000 × 10,000 pixels and 10,000 × 10,000 pixels, respectively. The images cover Lubei District, Guye District, Kaiping District, and Fengrui District. The images from ZiYuan-3 satellite with size of 24,500 × 20,000 is used to generate DTM.

### 3.2. Experimental Results and Analysis

#### 3.2.1. Accuracy of Faster R-CNN Trained Model

We performed the experiments on a computer with a 2.5 GHz Central Processing Unit (CPU) and a NVIDIA GeForce GTX 2080Ti Graphics Processing Unit (GPU). The memory sizes of CPU and GPU are 8 GB and 11 GB, respectively. The TensorFlow [33] deep learning framework was selected to train 861 Google map images of the BUAA-FFPP60 dataset. The pre-training model is the resnet101 [29] model trained on coco [30]. The number of training iterations is 170,000 and the learning rate is 0.001.

To evaluate the detection accuracy of the Faster R-CNN models, we test the trained model on test image of BUAA-FFPP60 dataset. When the detect target is true, the test result is a true positive (TP), and when the detect targets is false, the test result is false positive (FP). The false negative (FN) indicates the number of undetected true target in the image. Then, we can combine these into three metrics, precision (P), recall (R), and quality (Q):

(4) $$P=\frac{TP}{TP+FP}$$

(5) $$R=\frac{TP}{TP+FN}$$

(6) $$Q=\frac{TP}{TP+FP+FN}$$

For test samples, the precisions of working chimney, non-working chimneys, condensing tower, and non-condensing tower are 0.7210, 0.7326, 0.9482, and 0.9551, respectively. The recall rates are 0.8674, 0.8642, 0.9707and 0.9659 respectively. The qualities are 0.6451, 0.6629, 0.9423, and 0.9473, respectively.

#### 3.2.2. The Results from Faster R-CNN

After, we get the trained model. The Google images were input to the trained Faster R-CNN network. Due to the large area, the entire image is detected by window. The window size is 700 × 700 pixels and the step length is 500 pixels. The overlapped area in each step is as wide as 200 pixels, which is wide enough to prevent missing detection of chimneys at the edge of image. In order to detect more targets, we add an image enhancement method by adjusting the brightness and contrast ratio before Faster R-CNN detection. We also set a low network detection probability threshold, which is 0.3, to reduce the false negative and increase the recall rate.

**Figure 5:**
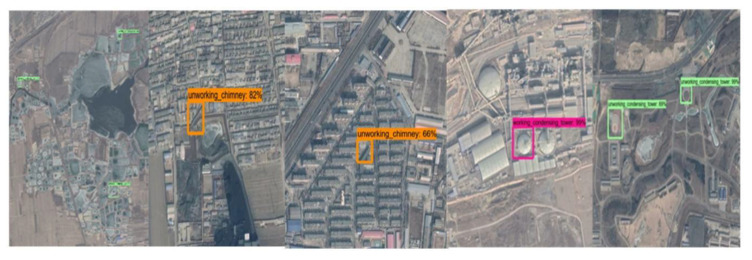
The false detection objects are divided into five categories: lake, road, architecture, tank, and other objects. The pink boxes represent working condensing tower, the green boxes represent non-working condensing tower, and the yellow boxes represent non-working chimney.

In order to analyze the detection accuracy, we divide the detection results into nine types: working chimneys, non-working chimneys, working condensing towers, non-working condensing towers, road, architecture, tank, lake, topography. Figure 5 shows some examples of false detection.

It can be found from Table 2 that the road and architecture are most likely to be mis-detected as chimneys, the number of which are 45 and 59 respectively. Condensing towers are most likely to be mixed up by tanks and lakes. The false detection rate of working chimneys, non-working chimneys, working condensing towers and non-working condensing towers are 0.5952, 0.5810, 0.8214, and 0.9166, respectively.

#### 3.2.3. The Results from Faster R-CNN, Elevation Filtering, and Main Direction Test

It can be found from Table 3 that by using the detection and test method—most of the false chimneys are removed. The false detection rate of working chimneys, non-working chimneys, working condensing towers and non-working condensing towers are significantly reduced to 0.0555, 0.0634, 0.1667, and 0.2, respectively. Meanwhile, only three non-working chimneys are mis-removed. That means after processing the true chimneys have been well retained.

#### 3.2.4. Discussion

Five false targets are shown in Table 4. The first line shows that cable tower is detected as non-working chimney. The cable tower is highly similar to chimney in both texture feature and three-dimensional structure. The main direction of the image slice is 30.19°, while the main direction of the whole image is 42.23°. This difference may be caused by some decorative or structural curves on the cable tower, which makes it not so straight in the image. However, similar loaded or decorative component is seldom attached on a chimney, so the true chimney is unlikely to be mis-removed. In the second line, a big tank is mistakenly detected as a working condensing tower. They are similar in height, so cannot be distinguished by only introducing the DTM. However, its aspect ratio, which is much smaller than true condensing tower, make the calculation of main direction after binarization unstable, leading to a large different with the image main direction. For the chimney like objects (including condensing tower), which has large aspect ratio, the main direction is determined by the pixel value distribution of wall. For those with low aspect ratio, such as the oil tank, the main direction is highly affected by the pixel distribution of its top cover. Therefore, the main direction test is also useful to distinguish some objects with different aspect ratio. In line 3, a complex scene with working and non-working chimneys, oil tanks, and steam vents is shown. There are only two chimneys in this image, one undetected working chimney in the red circle. The reason why the working chimney in the red circle remains undetected is that the two spatial analysis methods introduced in this paper are ineffective to reduce the false negatives. We think that the improvement in detection ability of neural network and completeness of the training dataset might be helpful. The detected non-working chimney is in the upper left corner. The rest of the detected objects are all false. The objects with lower height, including a steam vent, can be removed by DTM filtering. The main direction test can remove all false target in line 3 because the main directions of most interfering targets are randomly distributed except some high vertical objects. However, it is possible that the main direction of interfering target is coincidently consistent with the main direction of the image. Two examples show in line 4 and 5. The false targets cannot be removed by main direction test are mainly ground texture, shadows or structure that caused by overlapping.

The final evaluation indexes are shown in Table 5. The total target number (N) indicates the total chimneys in 3 images. The recall rates of four kinds of targets are 0.7727, 0.7662, 1, and 1, respectively. These values are much closed to the testing accuracies on BUAA-FFPP60 dataset. However, in practice, there is a large number of FPs, causing a very low precision. The original precisions are only 0.047, 0.4048, 0.2173, and 0.0833 for four kinds of target, respectively. After using two spatial analysis method, the FPs are largely removed. The precisions are increased to 0.9444, 0.9365, 0.833, and 0.8, respectively. The final qualities are 0.7391, 0.7108, 0.8333, and 0.8, respectively. The final qualities of working and nonworking chimneys are both significantly higher than the qualities calculated on testing samples. It can be concluded that the spatial analysis methods are very effective to increase the final precision and final quality.

In terms of category, chimneys have relatively low recall rate but high final precision. That is because the chimney is narrow in the image, and easily be interfered by noise, such as shadow, road, and build. Meanwhile, its unique contour makes it easy to distinguish with false chimney by spatial analysis method. In contrary, the condensing tower is easy to be detected by image-processing-based method, the Faster R-CNN, for its integrality appearance in image. Its relatively low final precision may partly result from the small number of samples.

## 4. Conclusions

In this paper, we use the Faster R-CNN to train the detection model on an open access dataset, BUAA-FFPP60. After the model is trained and tested, we used the model to detect the chimneys in three high-resolution remote sensing images of Google Maps, which is located in Tangshan city. The recall rates for working chimneys, non-working chimneys, working condensing towers, and non-working condensing towers are 77.27%, 76.62%, 100%, and 100%, respectively. However, the precisions for these targets are only 40.47%, 40.48%, 21.73%, and 8.3%, respectively. To increase the precision of detection, two spatial analysis methods, the DTM filtering and main direction test, are introduced to remove the false positive targets. The results show that more than 95% false chimneys can be removed, and the final precision of detection are 94.44%, 93.65%, 83.3%, and 80% respectively. There also exists a possibility that truly detected chimneys might be removed by these spatial analysis methods. However, in our experiment, only three non-working chimneys have been mistakenly removed. Therefore, DTM filtering and main direction tests are very effective methods to remove the false chimneys in detection results from Faster R-CNN. Although the two spatial analysis methods are very effective and robust to remove false positives, they are not useful to reduce the false negative. To reduce the false negative or increase the recall rate, we use an image enhancement method and a low Faster R-CNN threshold. We also suggest that further studies focus on more methods to reduce the false negatives, such as introducing more pre-processing, constructing new architecture of neural networks, and improving the completeness of the training dataset.

## Figures and Tables

**Table 1:** Threshold experiments.

Threshold	Chimneys	Condensing Tower	False Positive Targets
0	79	9	178
16	79	9	81
18	77	9	63
20	76	9	25
22	70	9	21
24	62	8	18

**Table 2:** Faster R-CNN detection result.

	Working Chimneys	Non-Working Chimneys	Working Condensing Towers	Non-Working Condensing Towers
working chimneys	17	1	0	0
non-working chimneys	0	62	0	0
working condensing towers	0	0	5	0
non-working condensing towers	0	0	0	4
roads	4	41	0	0
architectures	19	40	5	0
tanks	0	0	8	7
lakes	0	0	3	31
other objects	2	5	7	6
false detection rate	0.5952	0.5810	0.8214	0.9166

**Table 3:** Faster R-CNN+ elevation filtering + main direction detection result.

	Working Chimneys	Non-Working Chimneys	Working Condensing Towers	Non-Working Condensing Towers
Working chimneys	17	1	0	0
non-working chimneys	0	59	0	0
working condensing towers	0	0	5	0
non-working condensing towers	0	0	0	4
road	0	0	0	0
architecture	1	3	0	0
tank	0	0	1	1
lake	0	0	0	0
other objects	0	1	0	0
false detection rate	0.0555	0.0634	0.1667	0.2

**Table 4:** Examples of four-class detection method results. The pink boxes represent working condensing tower, the green boxes represent non-working condensing tower, the blue boxes represent working chimney, and the yellow boxes represent non-working chimney.

NO.	Faster R-CNN Detection	Combination of Faster R-CNN and Elevation Filtering Detection	Combination of Faster R-CNN and Main Direction Detection	Combination of Faster R-CNN and Elevation Filtering and Main Direction Detection
1	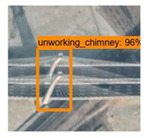	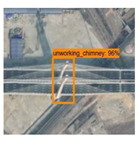	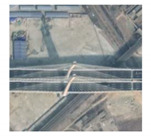	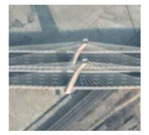
2	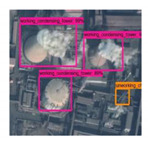	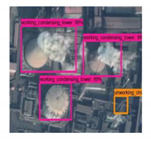	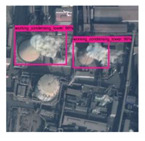	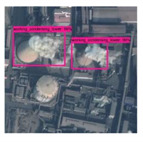
3	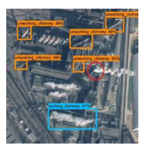	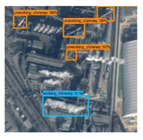	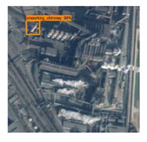	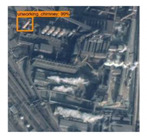
4	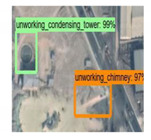	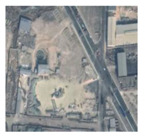	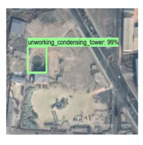	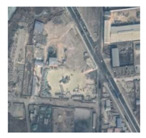
5	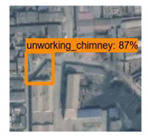	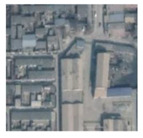	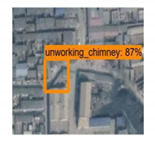	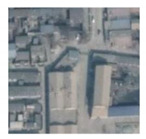

**Table 5:** The accuracy of the experiment.

Target Type	Working Chimneys	Non-Working Chimneys	Working Condensing Towers	Non-Working Condensing Towers
N	22	77	5	4
TP	17	62/59 *	5	4
FP	25	86	23	44
FN	5	18	0	0
Recall	0.7727	0.7662	1	1
Precision	0.4047	0.4048	0.2173	0.0833
Final Precision	0.9444	0.9365	0.833	0.8
Final Quality	0.7391	0.7108	0.8333	0.8

* Three non-working chimneys are mis-removed.
